# Label-free imaging of neurotransmitters in live brain tissue by multi-photon ultraviolet microscopy

**DOI:** 10.1042/NS20180132

**Published:** 2018-12-03

**Authors:** Barun Kumar Maity, Sudipta Maiti

**Affiliations:** Department of Chemical Sciences, Tata Institute of Fundamental Research, Homi Bhabha Road, Colaba, Mumbai 400005, India

**Keywords:** dopamine imaging, neurotransmitter dynamics, somatic exocytosis, Serotonin imaging

## Abstract

Visualizing small biomolecules in living cells remains a difficult challenge. Neurotransmitters provide one of the most frustrating examples of this difficulty, as our understanding of signaling in the brain critically depends on our ability to follow the neurotransmitter traffic. Last two decades have seen considerable progress in probing some of the neurotransmitters, e.g. by using false neurotransmitter mimics, chemical labeling techniques, or direct fluorescence imaging. Direct imaging harnesses the weak UV fluorescence of monoamines, which are some of the most important neurotransmitters controlling mood, memory, appetite, and learning. Here we describe the progress in imaging of these molecules using the least toxic direct excitation route found so far, namely multi-photon (MP) imaging. MP imaging of serotonin, and more recently that of dopamine, has allowed researchers to determine the location of the vesicles, follow their intracellular dynamics, probe their content, and monitor their release. Recent developments have even allowed ratiometric quantitation of the vesicular content. This review shows that MP ultraviolet (MP-UV) microscopy is an effective but underutilized method for imaging monoamine neurotransmitters in neurones and brain tissue.

## Introduction

Monoamine neurotransmitters play a pivotal role in the regulation of several processes, such as mood, emotion, reward, anger, aggression, appetite, sleep, and memory [1–3]. Alteration in their levels leads to various psychiatric and neurobiological disorders such as Schizophrenia, depression, Parkinson’s disease, and Alzheimer’s disease [4,5]. Therefore, mapping neurotransmitter distribution and dynamics in live systems is required not only to understand the neuronal circuits, but also to unravel the pathophysiology of many neurological diseases, and to improve the therapeutic strategy. However, it has been a major challenge to map them in live systems with high spatial and temporal resolution, especially in brain tissue. These molecules are of small size and they are not peptides, which prevents their visualization by the standard techniques of fluorescent protein labeling. The monoamines are related to aromatic amino acids, so they are fluorescent, but with typically low quantum efficiencies. More importantly, since these neurotransmitters absorb and emit in the UV region of the spectrum, optical microscopy becomes unsuitable, because exposure to UV light is toxic to living systems. In addition, high absorption and scattering of UV light neither allows penetration into brain tissue, nor is conducive for glass-based optical microscopy.

Various other experimental approaches have been employed to map these molecules in living systems. Positron emission tomography (PET) or single-photon emission computed tomography (SPECT) of radio-labeled dopamine and serotonin are widely used to obtain their distribution *in vivo* [6–8]. Another PET-based approach uses amount of free radio-labeled ligands, which competes with dopamine for receptor binding, as a measure of dopamine levels [9]. However, these approaches have been able to map monoamines in living systems with a resolution of ∼1 mm at best. An MRI-based method, which uses the heme domain of the bacterial cytochrome P450-BM3 as an MRI contrasting agent, has improved the resolution by ∼10-fold to image dopamine in the live brain [10]. But these techniques still lack resolution to follow the processes occurring at the subcellular level. There are a range of electrochemical techniques which can measure neurotransmitters at micro to nano-scale, but these are single point measurements, and do not provide images. For example, serotonin and dopamine have been measured with carbon fiber amperometry [11–13], and more recently with scanning electrochemical microscopy [14]. Optical microscopy with immunohistochemistry can probe these neurotransmitters at the cellular level, but it is not applicable to living systems. Direct UV light excitation has been used to image and to follow dynamics processes, such as exocytosis of monoamine neurotransmitters in live cells [15–18], but it affects cell heath significantly. To get rid of the harmful effects of UV excitation, single-photon excitable fluorescent false neurotransmitters (FFNs) have been designed, which can be selectively delivered vesicles containing monoamine transporters and can help follow their exocytosis from individual presynaptic terminals, but the signal is independent of the native concentration levels of the neurotransmitters [19–23]. There have been a few reports about monoamine sensors, including fluorescent ribonucleopeptide (RNP) [24] complexes, NIR emitting curcumin based turn-on sensors [25], and boronic acid-based fluorescent complexes [26,27]. But none of these is useful for intracellular analysis and imaging [28,29]. A fluorogenic detection method, inspired by the earlier work of Falck and Torp [30] and Falck et al. [31], has also been developed which can image monoamines in living cells [32]. This utilizes the fact that monoamine neurotransmitters, upon treatment with a small organic molecule *ortho*-phtaladehide (OPA), form a fluorescent compound, which emit in the visible region. Therefore, it allows visualization of the monoamine distribution in live systems without using harmful UV light. However, this does not give an absolute estimation of the concentration, since the yield of the reaction is unknown in cellular environments. In addition, the newly formed fluorescent complexes do not retain the native properties of the neurotransmitters, and likely perturb natural neurotransmission. Recent advances in mass spectroscopy imaging technique have enabled the generation of 3D maps of monoamine neurotransmitters at the subcellular level [33,34]. But this experiment is possible only with fixed cells. Also, this would not provide absolute concentration levels. Hence, it is important to explore benign, direct, and less perturbing approaches for the visualization and quantitative estimation of monoamines with high spatial and temporal resolution in live specimens.

Nature provides one significant advantage for imaging vesicular neurotransmitters, which can help overcome the low quantum efficiency of the fluorophores. This comes from their natural distribution: very high concentrations of the neurotransmitters (100’s of mM) are packed into the vesicles (diameter of synaptic vesicle: ∼40 nm, while for somatic vesicle: ∼50 to 100 nm) [35,36] making them bright punctate structures, which are almost the ideal type of objects for imaging. The other problem, the toxicity of UV light, can be mitigated by multi-photon (MP) optical microscopy. Two-photon microscopy can shift the required UV excitation wavelength to longer wavelengths [37], and higher order three-photon microscopy (3-PM) can in principle access these UV chromophores with IR excitation. Recently, other optical techniques have also been demonstrated which can image neurotransmitters with stimulated Raman scattering [38]. This is a powerful technique, but the signal from the membrane can overlap with the signal from neurotransmitters. In this review, we provide an overview of the MP microscopy techniques for imaging monoamine neurotransmitters such as serotonin and dopamine.

## Exciting monoamines with MP excitation

Till now, the most benign, label-free approach to image serotonin in live system has been to harness its UV fluorescence with MP excitation. Since, MP uses longer wavelength excitation, and excitation occurs only at the focal point with an excitation volume of ∼10^-16^ l, it causes limited damage to the biological systems and also provides high spatial resolution [39]. The transition probability for n-photon excitation is proportional to n^th^ power of the excitation intensity. The n-photon excitation phenomena can be approximately described by (eqn 1) mentioned below.

## Resolution for n-photon excitation

(1) $$\text{Rn}=\frac{0.6\lambda_{\text{n-photon}}}{NA\sqrt{n}}$$

Where λ_n-photon_ is the excitation wavelength for n-photon excitation and NA is the numerical aperture of the objective lens.

We note that λ_n-photon_ can be ∼nλ_1-photon_, which would make the resolution worse by the square root of *n*. However, typically λ_n-photon_ < nλ_1-photon_, and so the resolution penalty is much lower. However, the resolution is still worse than single photon excitation of the same fluorophore, which itself is bound by the diffraction limit. The diffraction limited resolution would be ≥200nm, which is not enough to resolve typical individual neurotransmitter vesicles. So in this review, we consistently use the term ‘vesicles/vesicular clusters’. However, one can still probe the exocytosis of individual subresolution-sized vesicles using MP microscopy from their host clusters, by following the stepwise decrease in fluorescence intensity of the clusters.

Though the advent of super resolution microscopy such as stimulated emission depletion (STED) microscopy, structured illumination microscopy (SIM), stochastic optical reconstruction microscopy (STORM), and photo-activated localization microscopy (PALM) has improved the resolution of optical microscopy beyond the resolution limit [40–42], it has not yet been possible to employ these techniques for label-free neurotransmitters imaging.

Two-photon excitation is the most prevalent MP technique. Tissue penetration is typically the best around near IR wavelengths [43], and for UV fluorophores, two-photon excitation would require a wavelength in the visible. To overcome this, a three-photon excitation scheme is more suitable, as it would require an IR excitation wavelength. However, three-photon excitation cross-sections are low, and this scheme may be expected to require higher power, but the more benign wavelengths may more than compensate for the photo-damage at the higher powers [44,45]. The action cross-section of serotonin with three-photon excitation is 6.4 × 10^−96^ m^−6^.s^2^.photon^−2^, while that for two-photon excitation is ∼80 mGM [39,46]. The comparison of two and three photon excitations showed that three-photon excitation with ∼100 fs pulses requires only a factor of approximately seven-times higher power than two photon excitation (see eqn 2 below for calculations) [46].

The rate of transition is given by [46,47].

(2) $$\gamma (\lambda )\;=\;\frac{\sigma_{n(\lambda )}}{R^{\left(n\;-\;1\right)}\tau^{\left(n\;-\;1\right)}}{\left(\frac{\lambda_{n\;}P_{ave}}{\pi \omega_{0n\;}^{2}hc}\right)}^{n}$$

Where σ_n_ is the molecular n-photon absorption cross-section, *P*_ave_ is the average power, R is the repetition rate of the laser, τ is the pulse width, ω_0_ is the beam waist (ω_0_ ∼ λ_n_/2NA, where NA is the numerical aperture of the objective lens), λ_n_ is the excitation wavelength, c is the velocity of light, *h* is the Planck’s constant.

An added complication for two-photon excitation is that typical femtosecond laser sources at this wavelength are more complicated and costly compared with their IR counterparts. However, for exciting at deeper UV wavelengths, such as that necessary for dopamine, one needs to resort to two-photon excitation, as we will describe later. Exploratory three-photon experiments were performed in the mid-nineties with these arguments in mind. The initial solution state measurements showed that it should be possible to image serotonin in living cells [48].

## Serotonin imaging in cells using 3-PM

To go from spectroscopy in the solution state to microscopy of a live specimen, one needs to make sure photodamage, light penetration and background are within reasonable limits [37]. Fortunately, all these factors are favorable for MP microscopy of these molecules, which makes serotonin imaging possible even in live brain slices at good penetration depths as we describe now [49,50].

**Figure 1:**
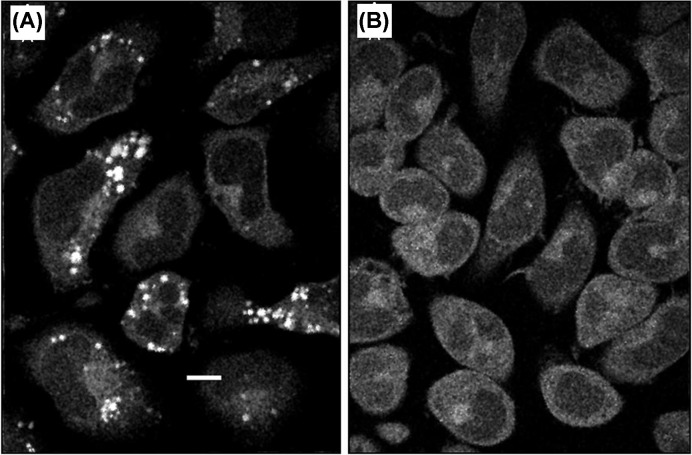
Images of RBL cells obtained from three-photon excited fluorescence of serotonin and other cellular components (**A**) Cells incubated in 250 µM serotonin for 6 h (imaged at a horizontal focal plane 3 µm above the coverslip.) (**B**) Control cells without serotonin incubation. Punctate fluorescence in (A) is caused by serotonin granules. (From Maiti, S.; Shear, J.B.; Williams, R.M.; Zipfel, W.R.; Webb, W.W. (1997) Measuring serotonin distribution in live cells with three-photon excitation. Science, **275** (5299), 530–532 [39]. Reprinted with permission from AAAS.)

The initial demonstration of three-photon excited UV fluorescence imaging of vesicular serotonin was in live rat basophilic leukemia (RBL-2H3) cells, which store serotonin in large somatic vesicles [39]. The externally added serotonin got actively transported into the cells, and subsequently to the secretory granules, which appeared as bright punctate structures. These were absent from the control images (see Figure 1A,B). However, whether this bright signal was due to native serotonin fluorescence or had contributions from other sources had to be verified. Spectral characteristics of the punctate fluorescence provided the initial proof. In addition, disappearance of these punctate structures after immunogenic stimulation supported that the signal was indeed from serotonin. The quantitative estimation of serotonin concentration was possible within the larger granules (>1 µm diameter) using the volume of granules measured from 3D images and using the fluorescence intensity of serotonin solution of known concentration as a calibrant [39]. Later, Williams et al. [51], also measured serotonin in live cells using this technique, and by comparison with HPLC, proved that it can be used for quantitating serotonin in live specimens.

**Figure 2:**
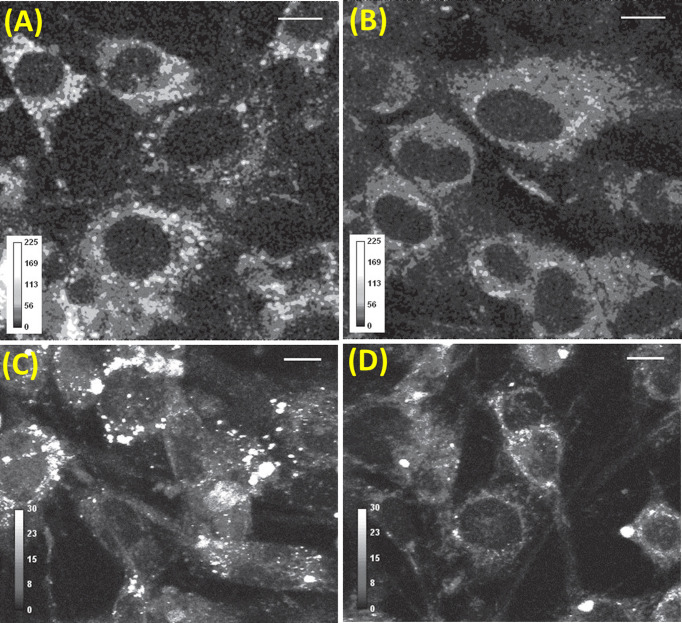
Intracellular content and vesicular concentration change as cells differentiate (**A**) Differentiated cells contain more serotonin and many more vesicles compared with (**B**) their undifferentiated counterparts, as shown in these gray scale intensity coded images. Scale bar = 10 µm. Serotonin synthesis inhibition with 90 µM PCPA reduces serotonin content in differentiated cells (**D**) compared with control (**C**). (From Balaji, J.; Desai, R.; Kaushalya, S.K.; Eaton, M.J.; Maiti, S. (2005) Quantitative measurement of serotonin synthesis and sequestration in individual live neuronal cells. *J. Neurochem.*, **95**(5), 1217–1226. Reprinted with the permission from John Wiley and Sons.)

Now, potentially the most crucial application of the ability to measure serotonin at subcellular levels is in the context of chemical neurotransmission. RBL cells have a large amount of serotonin in somatic vesicles, and it was not obvious whether this same approach would be able to image serotonin in neurones. The small neurotransmitter vesicles were expected to be mostly located in the synaptic regions. Chemical neurotransmission consists of three major steps: synthesis, sequestration into vesicles, and exocytosis. A measurement of the total serotonin content (both sequestered and non-sequestered) is important to understand the first two steps, and also the effect of psychoactive agents such as amphetamines in a quantitative manner. On the other hand, physiological effects for example, if something makes the vesicles leaky (suggested as a pathway of action for amyloid β in Alzheimer’s disease [52], and also for the action of amphetamines) are likely to be mediated through alterations in the content of neurotransmitters sequestered into vesicles [53]. It can also be important in other contexts, e.g. during the differentiation of stem cells into serotonergic neurones. The serotonin content changes gradually as the cells differentiate into serotonergic neurones [54]. Therefore, probing the concentration of serotonin at the vesicular level would be helpful in understanding both normal and diseased states of the brain. Balaji et al. quantitated intra-vesicular serotonin content in serotonergic neuronal cell line, RN46A [55] using MP microscopy [56]. These cells are derived from rat serotonergic neurones, and contain a temperature-sensitive mutation which allows proliferation at lower temperatures (33°C) and differentiation at higher temperatures (39°C). The large amount of vesicular serotonin contained in the soma was a surprise from the initial images. It had been suggested earlier by some researchers that the monoaminergic neurones contain somatic vesicles which are exocytosed from the soma itself [57–60], but direct proof was lacking. These 3-PM results provided a direct proof of this phenomenon. Additionally, these experiments showed the relationship between synthesis and sequestered content. Upon temperature-induced differentiation, total serotonin content per cell increases, and when its synthesis is inhibited in differentiated cells, total content decreases, as may be expected. However, in contrast, the amount of sequestered proportion remained similar even after the chemical inhibition of synthesis (see Figure 2). Therefore, the total content is not a reliable indicator of the sequestered amount [56]. We note that the optical technique required for MP microscopy of serotonin can be easily implemented in a MP microscope and is well-described in Kaushalya et al. [61,62].

Is substantial amount serotonin released from the somatic vesicles through somatic exocytosis? Is it substantial in comparison with synaptic release? To answer these questions, Kaushalya et al. (2008) followed the dynamics of serotonergic vesicles/vesicular clusters using 3-PM[63]. They demonstrated that the amount of serotonin that gets released through somatic exocytosis under K^+^-induced membrane depolarization of a raphe neurone is comparable with the amount of total serotonin content in all the synaptic vesicles. Exocytosis was verified by labeling the vesicular traffic by the membrane labeling dye FM1-43 [63,64].

## Quantitation of serotonin content

The exact concentration of serotonin cannot be estimated directly from fluorescence imaging, because of uncertainties of quantum efficiency, local environment, possible presence of quenchers, and collection efficiency [65]. A major additional problem is that a large proportion of vesicles has a size less than the minimum resolution [66], and therefore the apparent brightness depends on both the vesicular volume and the concentration of serotonin in these vesicles.

Amperometry can estimate serotonin concentration from the current generated at the electrode due to the oxidation of serotonin released from individual vesicles, but it cannot do so for intracellular vesicles [35,66,67]. On the other hand, under some assumptions, three-photon imaging can give an estimate of the concentration in the vesicles. For this estimation, it was assumed that the vesicles are spherical in shape and there was a near-constant concentration of serotonin irrespective of the size of the vesicles [56]. The size versus expected fluorescence intensity was then simulated and compared with the experimentally obtained results using a linear scaling factor as the only free parameter. Under these assumptions, the concentration of serotonin within these vesicles was estimated to be ∼400 ± 50 mM, which is somewhat high compared with the estimation (∼270 mM) made by Bruns et al. (2000) [35] in leech neurones. However, good agreement of the simulated data with the observed data indicated that the assumptions are self-consistent [56]. In any case, it would be desirable to improve the method for measuring the concentration of subresolution objects within living systems. We discuss this next.

## Quantitative estimation of serotonin concentration by ratiometry

**Figure 3:**
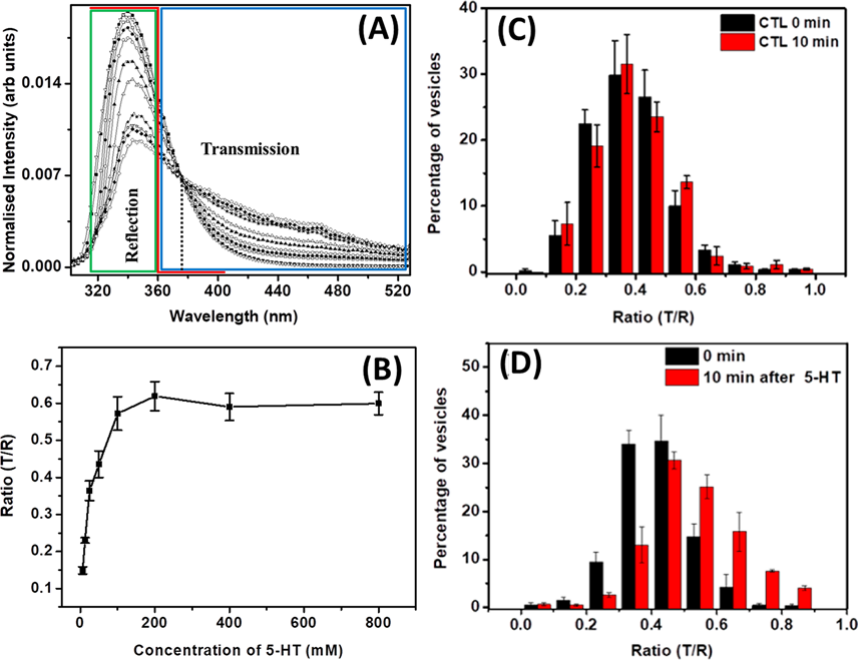
(**A**) Area-normalized fluorescence emission spectra of serotonin at different concentrations (at 60 µM, 1, 12.5, 25, 50, 100, 200, 300, 400, 500, and 600 mM, respectively). The iso-emissive point at 376 nm is shown with a dotted vertical line. Solid red line schematically shows the spectral characteristic of the UV dichroic (360 nm long pass). (**B**) Three-photon fluorescence intensity ratio of transmission (T) and reflection (R) obtained from serotonin solutions as a function of concentration. The reflected signal is collected from the region indicated by the green box in (A), while the transmitted signal is collected from the region indicated by the blue box in (A). (**C**) Histogram of the mean ratios of signals collected in the transmission and reflection channels (T/R) at 0 and 10 min after vehicle treatment. (**D**) Histogram of the mean T/R ratio after 0 and 10 min after treatment with externally added serotonin solution (400 μM) (error bar = S.E.M.). (A) Reprinted from Nag S., Balaji J., Madhu P.K. and Maiti, S. (2008) Intermolecular association provides specific optical and NMR signatures for serotonin at intravesicular concentrations. *Biophys. J.*, ** 94**, 4145–4153, copyright (2008) with permission from Elsevier. (B–D) Reprinted with permission from Das, A.K.; Maity, B.K.; Surendran, D.; Tripathy, U.; Maiti, S. (2017) Label-free ratiometric imaging of serotonin in live cells. *ACS Chem. Neurosci.*, **8**(11), 2369–2373 [71]. Copyright (2017) American Chemical Society.

In fluorescence microscopy, a reliable estimate of concentration is frequently obtained by ratiometric measurements. In such cases, the fluorescent dye has spectral characteristics which change with the concentration of the analyte. Therefore the fluorescence intensity is measured at two or more excitation/emission wavelength, and the ratio of intensity at two different wavelengths can report the analyte concentration [68,69]. It is not obvious how such estimation can be possible with label-free imaging, such as that of serotonin. However, high concentration behavior of serotonin provides a way to do this. It has already been reported that the shape of the serotonin spectrum changes as a function of its concentration [70]. At low concentration (∼9.4 mM), it shows a single emission maximum at ∼340 nm. But with increase in concentration, a new peak arises at 425 nm [46] (see Figure 3A). Nag et al. (2008) [70] have shown that the fluorescence lifetime and also the NMR characteristics differ at higher concentrations, which clearly suggest the formation of a new oligomeric species. This provided an opportunity to perform ratiometric imaging for serotonin, as a function of its concentration. As the spectral features of the monomer and the oligomers are different, the ratio of fluorescence intensity at the two different wavelengths could act, in principle, as an internal ruler for serotonin concentration. Das et al. (2017) [71] executed this approach in RN46A cells derived from rat neurones to measure the vesicular concentrations of serotonin using two channel 3-PM. The concentration-dependent calibration curve was generated by dividing the signals into two channels (with appropriate filters) (see Figure 3B). Now, the ratio of fluorescence signals from the two channels report the vesicular serotonin concentration, and the solution state calibration curve can be used as a reference [72]. When serotonergic RN46A cells were exposed to externally added serotonin, the distribution of the ratios shifted toward higher values and suggested an increased uptake of serotonin (see Figure 3C,D). The values of the ratios indicated that the vesicles on an average had ∼41 mM before and ∼84 mM after incubation with external serotonin. However, these values are low compared with the values estimated earlier. This indicates the need for a correction factor to take care of the background auto-fluorescence. However, this is still a robust technique to follow relative changes during vesicle refilling or during specific perturbations of vesicular concentrations by chemical agents such as amphetamines [53].

## High-resolution mapping of serotonin in the brain

**Figure 4:**
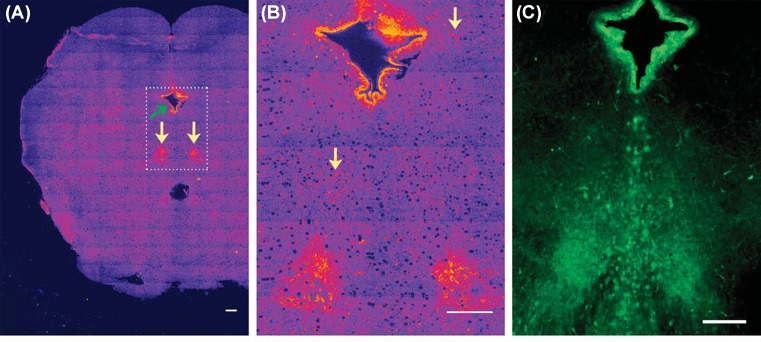
Mapping serotonin distribution in live rat brain slice (**A**) Three-photon excited serotonin distribution in a brain slice containing the raphe nuclei (marked by yellow arrow). The image is made by stitching 160 high-resolution images. (**B**) Zoomed up view of the rectangular region [dashed lines in (A)] containing the raphe and the ependymal region. Arrows here mark some non-raphe regions. (**C**) Epifluorescence image of serotonin immunofluorescence in a similar region of a brain slice (Reprinted with permission from Kaushalya, S.K.; Nag, S.; Ghosh, H.; Arumugam, S.; Maiti, S., (2008) A high-resolution large area serotonin map of a live rat brain section. *Neuroreport*, **19**, 717–721 [50]). Copyright(2008) Wolters Kluwer Health, Inc.

While direct imaging showed that a major amount of serotonin is released from the somatic vesicles, these experiments were carried out in cultured neurones. Therefore, these experiments did not prove that somatic storage and release can constitute a substantial component of serotonergic neurotransmission in the brain. The dorsal raphe (DR) nuclei contain the cell soma of the majority of serotonergic neurones, which send projections to most parts of the brain from there. If the soma contains a major amount of the serotonin even in the brain, then the raphe region, which is anatomically identifiable, should appear bright in a brain-wide image. And if this serotonin is releasable, then this somatic brightness should go down upon depolarization. However, probing this would require one to image at least one optical slice of the whole rat brain, with enough resolution to locate serotonin in intracellular compartments. Also, the imaging technique should be benign enough to allow the neurones to respond to subsequent depolarization. Kaushalya et al. [50] showed that three-photon imaging can be used to map serotonin in one whole slice of a rat brain at high resolution, and the release can be subsequently monitored. The images clearly showed that the raphe region contains a large amount of releasable serotonin. The ependymal region also contained a considerable amount of serotonin (Figure 4). The total integrated serotonin content in the raphe region was ∼0.87-times of that present in the ependymal region. However, under depolarization conditions, raphe region released more serotonin compared with the ependymal region. This proved that somatic serotonin can indeed be a major factor in serotonin release in the brain. Also, it is interesting to note that the total content in a tissue does not linearly correlate with the releasable content [50,63].

Later Colgan et al. (2012) [73] has also mapped vesicular serotonin in the DR neurone dendrites using 3-PM, where the vesicular clusters appear to be spindle shaped. Release of serotonin from these vesicles is not controlled by the action potentials, unlike the release from the soma and the terminals. Such unique way of controlling serotonin release has important implications for DR physiology [73].

## Probing the dynamics of somatic exocytosis using MP microscopy

**Figure 5:**
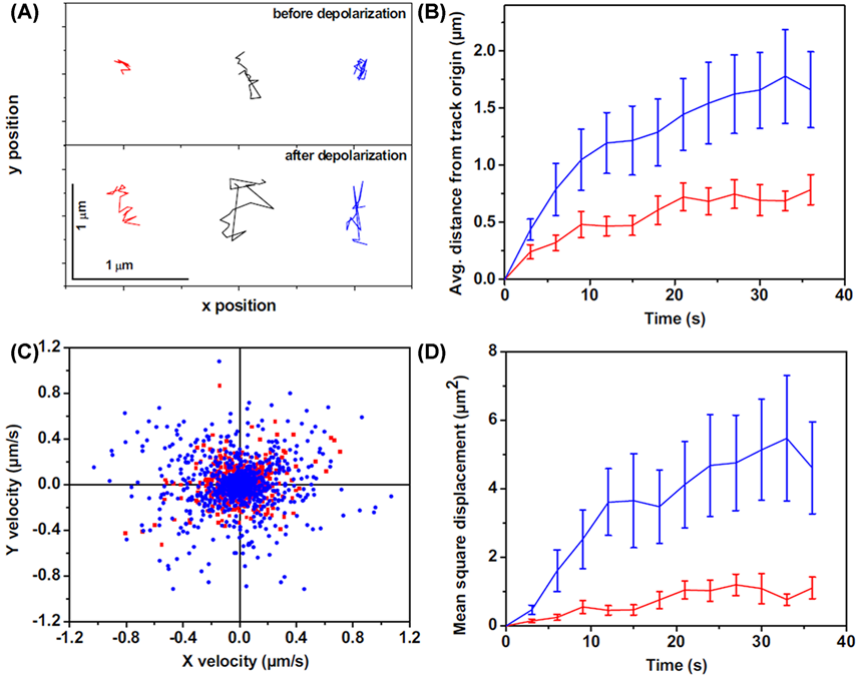
The dynamics of somatic vesicles pre-and post-depolarization Three-photon excitation microscopy shows dynamics of somatic serotonergic vesicles (or unresolved vesicular clusters) in the cell body and in the processes before and after KCl-induced depolarization. (**A**) Individual tracks of a few serotonin fluorescent spots before (top) and after (bottom) depolarization. In (**B**–**D**), red and blue denote measurements performed before and after depolarization, respectively. (B) Average displacement from the track origin compared with time. (C) Velocity distribution obtained from the tracks. (D) MSD compared with time (error bars correspond to S.E.M.). (Adapted from Sarkar, B.; Das, A.K.; Arumugam, S.; Kaushalya, S.K.; Bandyopadhyay, A.; Balaji, J.; Maiti, S. (2012) The dynamics of somatic exocytosis in monoaminergic neurons. *Front. Physiol.*, **3**, 414).

How do the somatic vesicles move from their resting locations to the plasma membrane before exocytosis? 3-PM allows the tracking of the trajectories of the vesicles to the plasma membrane in response to the stimulation of living neurones. While Kaushalya et al. (2008) [63] followed exocytosis dynamics including vesicle/vesicular clusters movement, it was more precisely quantitated by Sarkar et al. [74]. They looked at the somatic exocytosis process in serotonergic neurones from raphe nuclei of the brain using 3-PM and tracked the movement of individual vesicles (or unresolved vesicular clusters) during exocytosis. In the resting state, the serotonergic vesicles/vesicular clusters do not show much movement from their location. Upon membrane depolarization, vesicles/vesicular clusters become more dynamic, with a mean square displacement (MSD) increasing from ∼0.04 to ∼0.30 µm^2^/s. Interestingly, this movement only occasionally results in exocytosis. The pre-exocytosis movement does not have any specific directionality, which suggests a random diffusion or transport. The range of distance that they traverse in this phase is typically a few microns, which is larger than what they traverse before depolarization (Figure 5). Hence, it appears that depolarization de-tethers the neurotransmitters’ vesicles from their storage locations and allows them to move by a process of constrained diffusion. However, the movement can be too fast to follow using the MP scanning microscopy, as the scanning speed is limited to ∼1 s per optical slice if one wants to obtain an image with a good signal to noise. To overcome this limit, total internal reflection fluorescence microscopy was used to follow the exocytosis dynamics in the same cells using single photon marker dyes. The results indicated that somatic serotonin-containing vesicular clusters stay in the vicinity of the membrane for only ∼25 ms before exocytosis [74]. While this process is much slower than synaptic exocytosis [75], it is much faster than the exocytosis of hormones. In fact, this is the fastest somatic exocytosis observed so far in mammalian cells.

## Serotonin during embryonic development and stem cell differentiation

Serotonin is present early in the embryos of many species, where evidence suggests its participation on important role in various early developmental processes [76–79]. Therefore, the ability to estimate serotonin distribution can be important for following developmental processes. The level of serotonin in the embryo has been measured using anti-serotonin antibodies, but this is not very quantitative, and does not provide information about live samples [80]. Basu et al. [81] measured the serotonin distribution in pre-implantation mouse embryos, where the brain has not yet formed, using 3-PM. The images appear to have punctate features of average size ∼1.3 µm^2^, which are not necessarily vesicular bodies. Immunohistochemistry using anti-serotonin antibody, and mass spectroscopy of the embryo extract showed that these punctate features are indeed due to the presence of serotonin. Using serotonin solutions as reference, they estimated the serotonin concentration in these puncta to be 100–400 mM.

Later, 3-PM was employed to study the differentiation of human-induced pluripotent stem cells (hiPSCs) cells into serotonergic neurones. It enabled Kumar et al. [82] to follow the synthesis and sequestration of serotonin in differentiating hiPSC cells over several weeks. Even in these cells, serotonin vesicles/vesicular clusters appear as punctate structures, which are distributed throughout the cell body. Upon KCl-induced depolarization, a number of vesicles/vesicular clusters disappeared from the cell body, presumably through exocytosis, and the extent of this release changed with the maturation of the neurones. The ability to look at these dynamic changes offers a powerful way of investigating the development of iPSCs into serotonergic neurones and has considerable clinical significance [82].

## Dopamine imaging using MP microscopy

Dopamine is another important monoamine neurotransmitter, but it emits in a deeper UV region (excitation: ∼270 nm, emission: <320 nm) compared with serotonin. This introduces an additional difficulty, because the optics used in the conventional epi-fluorescence microscopes for MP microscopy has poor transmission in this region. Balaji et al. (2002) [83] had demonstrated that an efficient way to collect the UV fluorescence is to use a lens-free detection geometry. They also compared the collection efficiency with epi-fluorescence collection. To get rid of any intervening optical elements in the collection path, the photomultiplier tube detector was placed right above the objective lens of an inverted microscope, at the minimum possible distance from the specimen. Their geometry yielded an estimated effective NA of 0.64, which is within a factor of two of the best water immersion objective. The major requirement of this non-epifluorescent mode of collecting signals is to block the high power excitation light. This requirement was fulfilled in this case by using an absorptive glass based filter, which effectively blocked the green light. The available glass based filters unfortunately do not block IR light as efficiently, and so the only practical solution is to perform two-photon microscopy using 540 nm excitation light obtained from an optical parametric oscillator. The comparison between non-epi and epi-collection geometries showed that the collection efficiency for dopamine is ∼68-folds higher in the straight geometry compared with the epi-fluorescence geometry, and this factor is also greater than one for other tested UV emitting chromophores such as serotonin and norepinephrine [83]. Instead of placing the detector as close as possible to the sample, a quartz condenser lens can also be used in the collection path [84]. Use of quartz objective lens for the epi-detection might be another alternative, but much lower optical quality of these objective lenses compared with their glass based counterparts, and the presence of other glass based elements in the optical path can cause problems [83]. Therefore, non-epi collection is perhaps the most effective way to image the deeper UV emitting chromophores.

**Figure 6:**
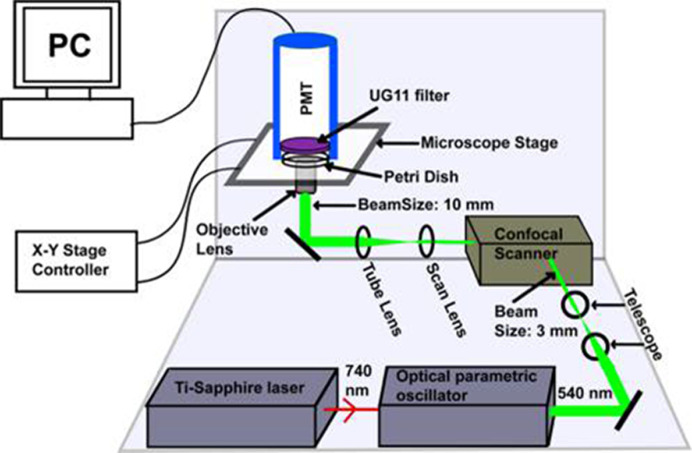
Schematic representation of the optical setup for dopamine imaging (not to scale) An optical parametric oscillator synchronously pumped by mode-locked tunable Ti-Sapphire laser at 740 nm produces 540 nm ∼100 fs pulses. The collimated beam (3 mm in diameter, achieved using a telescope setup) is routed through a confocal scan-box to an inverted microscope equipped with a 60× water immersion objective lens (numerical aperture, NA = 1.2). The beam diameter is 10 mm at the back of the objective lens and has an average power of 30 mW. The emission is collected in the forward direction, with an external photomultiplier tube (PMT) placed directly above the sample. A pair of special glass based absorptive filters (UG11) is placed in front of the PMT which transmits the mid-UV fluorescence but efficiently blocks the 540 nm excitation light. (Reprinted with permission from Sarkar, B.; Banerjee, A.; Das, A.K.; Nag, S.; Kaushalya, S.K.; Tripathy, U.; Shameem, M.; Shukla, S.; Maiti, S. (2014) Label-free dopamine imaging in live rat brain slices. *ACS Chem. Neurosci.*, **5**(5), 329–334 [49]. Copyright (2014) American Chemical Society.)

**Figure 7:**
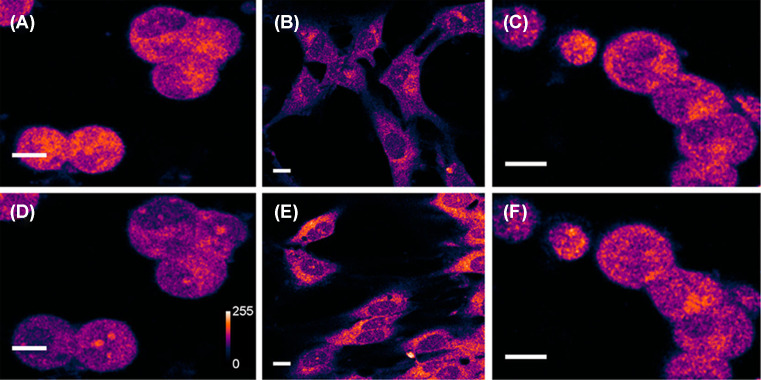
Two-photon auto-fluorescence of dopamine in dopaminergic MN9D cells and in glia (**A,D**) Collection of MN9D cells before (A) and 15 min after (D) treatment with 100 μM of PCA. The signal intensity decreases by 34 ± 5%. Vehicle-treated control cells are shown under identical imaging conditions in (**C**) (before) and (**F**) (after). The control cells show 1 ± 3% increase in the same period. (**B,E**) Glia treated with vehicle (B) and 10 mM dopamine for 4 h (E). Fluorescence intensities are false color coded. For (B,E), the brightness is 3× enhanced. Scale bar = 10 μm. (Reprinted with permission from Sarkar, B.; Banerjee, A.; Das, A.K.; Nag, S.; Kaushalya, S.K.; Tripathy, U.; Shameem, M.; Shukla, S.; Maiti, S. (2014) Label-free dopamine imaging in live rat brain slices. *ACS Chem. Neurosci.*, **5**(5), 329–334 [49]). Copyright (2014) American Chemical Society.)

Sarkar et al. [49] employed this non-epifluorescence collection strategy to visualize native dopamine fluorescence in living cells in culture, and also in rat brain slices (see Figure 6). Two-photon images of dopaminergic cells MN9D [85,86] appeared bright, with the darker regions representing the nucleus. However, there were no punctate features comparable with what was observed for serotonin in RN46A cells. Cultured glia cells, which are not expected to have dopamine, show much less brightness. However, the brightness of the glial cells increases when they are incubated with dopamine for some time. When the MN9D cells were treated with *para*-chloroamphetamine (PCA), a powerful monoamine releasing drug, brightness goes down significantly. The vehicle-treated control set of cells exhibit negligible change in brightness (see Figure 7). In addition, the identity of the imaged fluorophore was validated using HPLC coupled MS, where the samples containing extracts of MN9D were mixed with a known amount of isotopically labeled dopamine for quantitation. The results confirmed the presence of dopamine commensurate with the signal observed in microscopy. Together these results confirmed that the signals are indeed mostly contributed by dopamine, and not by other UV emitting chroromophores present in the cells. If the background can be estimated better, the method will be able to provide a robust estimation of dopamine levels in live specimens [49]. Unfortunately, dopamine does not display any robust concentration-dependent change in the emission spectrum like serotonin, so ratiometry cannot be performed.

Recently, dopamine neurotransmission in live dopaminergic cells has been mapped with high spatial and temporal resolution by using MP excitation using an NIR emitting nanosensor array of DNA wrapped single-walled carbon nanotubes (SWCNs). The cells grown on top of these nano-arrays were stimulated externally, and the released dopamine changed the fluorescence property of the SWCNs when it came in contact with it. The time-dependent intensity changes surrounding the cells can provide the directionality of dopamine efflux [87]. Similar approach was also demonstrated earlier, but using a limited number of microeletrodes per array for estimating catecholamine release directions and dynamics [88–90]. This is a powerful technique to estimate the exocytosed dopamine and to understand how dopamine signaling occurs. However, it cannot measure the intracellular dopamine levels. Combination of MP imaging and nano-array sensing, if possible in future, may be a rather powerful technique for probing dopaminergic signaling.

Imaging dopamine distribution in live brain slices is more challenging than imaging in cultured neurones due to the strong absorbance and scattering of the emission signal by the intervening tissue. Despite these difficulties, dopamine was successfully imaged in cultured rat brain slices. The images showed bright punctate structures mostly near the Substantia Nigra (SN) region, which is known to be enriched with dopaminergic neurones. There were fewer punctate features in the area outside the nigra. These punctate features are likely due to the neuronal vesicles or vesicular clusters containing dopamine at high concentration [49]. The punctate structures are distributed throughout the cell body and also in the primary neurite of the neurone. This configuration can image dopaminergic neurones in live brain tissues till a depth of at least 25 µm without losing much resolution and signal intensity [49].

## Summary

MP microscopy is a powerful technique for quantitative high resolution imaging of monoamine neurotransmitters, such as serotonin and dopamine, in live specimens in a benign, label-free manner. The technique is easy and effective, but its potential remains underutilized. Combining this technique with other complementary ones, such as carbon fiber amperometry, can yield fundamental insights about the monoaminergic signaling process, and a deeper understanding of the effects of pharmacological agents (and drugs of abuse) on these processes.

## Abbreviations

3-PM: three-photon microscopy
DR: dorsal raphe
hiPSC: human-induced pluripotent stem cell
HPLC: High performance liquid chromatography
MP: multi-photon
MS: Mass spectroscopy
NIR: Near Infrared
PET: positron emission tomography
SN: Substantia Nigra
SWCN: single-walled carbon nanotube
